# Physiology, Biochemistry, and Pharmacology of Transporters for Organic Cations

**DOI:** 10.3390/ijms22020732

**Published:** 2021-01-13

**Authors:** Giuliano Ciarimboli

**Affiliations:** Experimental Nephrology, Department of Internal Medicine D, University Hospital Münster, 48149 Münster, Germany; gciari@uni-muenster.de

This editorial summarizes the 13 scientific papers published in the Special Issue “Physiology, Biochemistry, and Pharmacology of Transporters for Organic Cations” of the *International Journal of Molecular Sciences*. In this Special Issue, the readers will find integrative information on transporters for organic cations. Besides reviews on physiology, pharmacology, and toxicology of these transporters [1,2,3], which offer a concise overview of the field, the readers will find original research work focusing on specific transporter aspects.

Specifically, the review “Organic Cation Transporters in Human Physiology, Pharmacology, and Toxicology” by Samodelov et al. [1] summarizes well the general aspects of physiology, pharmacology, and toxicology of transporter for organic cations. The other review “Organic Cation Transporters in the Lung—Current and Emerging (Patho)Physiological and Pharmacological Concepts” by Ali Selo et al. [2] focuses on these aspects of transporters for organic cations in the lung, an important but often neglected field.

In the paper “Systems Biology Analysis Reveals Eight SLC22 Transporter Subgroups, Including OATs, OCTs, and OCTNs”, by performing a system biology analysis of SLC22 transporters, Engelhart et al. [4] suggest the existence of a transporter–metabolite network. They propose that, in this network, mono-, oligo-, and multi-specific SLC22 transporters interact to regulate concentrations and fluxes of many metabolites and signaling molecules. In particular, the organic cation transporters (OCT) subgroup seems to be associated with neurotransmitters and the organic cation transporters novel (OCTN) subgroup seems to be associated with ergothioneine and carnitine derivatives. Transporters of the solute carrier (SLC) 22 family may work together with transporters from other families to optimize levels of numerous metabolites and signaling molecules involved in organ crosstalk and inter-organismal communication, according to the remote sensing and signaling theory.

In the other paper by Engelhart et al. “Drosophila SLC22 Orthologs Related to OATs, OCTs, and OCTNs Regulate Development and Responsiveness to Oxidative Stress”, an evolutionary analysis of putative SLC22A transporter orthologs in *Drosophila melanogaster* was performed [5]. At least 4 fruit fly transporters, probably involved in the handling of reactive oxygen species, seem to be SLC22 orthologues.

Neurotransmitters such as serotonin are important endogenous organic cations. Interestingly, the anesthetic drug ketamine has an antidepressant action. In the paper “Serotonin Transporter and Plasma Membrane Monoamine Transporter Are Necessary for the Antidepressant-Like Effects of Ketamine in Mice”, Bowman et al. investigated whether this effect of ketamine is due to an influence on extracellular serotonin concentration [6]. They demonstrated that ketamine decreases serotonin clearance from the Cornu Ammonis (CA) 3 region of the murine hippocampus in vivo, probably by acting on the serotonin transporters (SERT) and the plasma membrane monoamine transporter (PMATs).

Since organic cation transporters are normally expressed in well-differentiated cells and they can be involved in the cellular uptake and/or efflux of chemotherapeutic drugs, their expression level may be related to the prognosis of cancer clinical outcome. In the communication “Identification of Prognostic Organic Cation and Anion Transporters in Different Cancer Entities by In Silico Analysis”, Bayram Edemir [7] analyzed the relationship between expression of transporter mRNA and survival probability. To do this, he used data provided by The Cancer Genome Atlas (TCGA), where next-generation RNA-sequencing data for the most common tumor entities in a cohort which comprises more than 12,800 samples derived from 17 different tumor types are enclosed. In most cases, the expression level of organic cation transporters had a favorable prognostic value, suggesting that, in these cancers, tumor cells still show a certain grade of differentiation and/or better uptake of chemotherapeutic drugs.

Acute regulation of transporter activity can change the exposure of the body to drugs. While the regulation of organic cation transporters is well known, there is only scarce information on Multidrug and Toxin Extrusion Transporters (MATE) regulation. This aspect of MATE function was analyzed in detail in the paper “Rapid Regulation of Human Multidrug and Extrusion Transporters hMATE1 and hMATE2K” by Kantauskaité et al [8]. MATE activity was regulated both in uptake and in the efflux transporter configuration by several protein kinases. Some regulation pathways are common to those previously observed for OCTs, suggesting that there is the possibility to regulate hepatic and/or renal secretion of organic cations. 

The activity of renal OCT2 and MATE transporters was also regulated by the transcription factor Farnesoid X receptor (FXR), for which activation increased the expression and activity of the transporters, as demonstrated in the paper “Farnesoid X Receptor Activation Stimulates Organic Cations Transport in Human Renal Proximal Tubular Cells” by Wongwan et al. [9]. On the other side, peroxisome proliferator-activated receptor alpha (PPAR-α), which is also a transcription factor, increases OCT2 and decreases MATE1 renal expression, as demonstrated in the paper “PPAR-Deletion Attenuates Cisplatin Nephrotoxicity by Modulating Renal Organic Transporters MATE-1 and OCT-2” by Freitas-Limaet al. [10]. The authors demonstrated also that genetic deletion of PPAR-α was able to protect against cisplatin-induced nephrotoxicity by decreasing OCT2 expression (OCT2 is an uptake transporter for cisplatin) and by increasing MATE1 expression (MATE1 is considered to be the secretion transporter of cisplatin).

Another mechanism by which transporter expression and function can be altered is by mutations due to the presence of single nucleotide polymorphisms (SNPs). The review “The Impact of Genetic Polymorphisms in Organic Cation Transporters on Renal Drug Disposition” by Zazuli et al. [3] illustrates the impact of OCT genetic polymorphisms on renal drug disposition and kidney injury, their clinical significances, and how to personalize therapies to minimize the risk of drug toxicity.

Focusing on the potential pharmacological role of OCT, in the paper “Tofacitinib and Baricitinib Are Taken up by Different Uptake Mechanisms Determining the Efficacy of Both Drugs in RA”, Amrhein et al. [11] demonstrates that the tyrosine kinase inhibitor tofacitinib, which is approved and recommended by the European League Against Rheumatism for the treatment of rheumatoid arthritis (RA), is transported by MATE1. The expression of MATE1 is reduced under inflammatory conditions and in synovial fibroblasts from RA patients, suggesting that tofacitinib cannot exit the cells and, for this reason, has a favorable impact as RA therapeutic drug.

Rodents are used as a preclinical model to study the biological effects of drugs and xenobiotics. The paper “Functional and Pharmacological Comparison of Human, Mouse, and Rat Organic Cation Transporter 1 toward Drug and Pesticide Interaction” by Floerl et al. [12] investigates the interaction of several drugs and pesticides with mouse, rat, and human OCT1. They show that, in general, rodent and human OCT1 have the same type of interaction with these substances. However, species-specific differences can exist and should be investigated for new molecular entities. Similarly, focusing on the muscarinic receptor antagonist trospium chloride, in the investigation on “Trospium Chloride Transport by Mouse Drug Carriers of the Slc22 and Slc47 Families”, Gorecki et al. [13] demonstrated that trospium is transported with similar characteristics by mouse and human OCT1, OCT2, and MATE1.
